# The feasibility of using a multivariate regression model incorporating ultrasound findings and serum markers to predict thyroid cancer metastasis

**DOI:** 10.3389/fendo.2024.1461865

**Published:** 2024-11-18

**Authors:** Hong Li, Lanli Zhang, Yanbing Wang, Shengju Tong, Yang Shi, Shengnan Lu, Yanling Bu

**Affiliations:** ^1^ Department of Ultrasound, The Third Affiliated Hospital of Qiqihar Medical University, Qiqihar, China; ^2^ Computer Center, The Third Affiliated Hospital of Qiqihar Medical University, Qiqihar, China; ^3^ Department of General Surgery, The Third Affiliated Hospital of Qiqihar Medical University, Qiqihar, China

**Keywords:** ultrasound findings, serum markers, thyroid cancer, distant metastasis, multivariate regression model

## Abstract

**Objective:**

This study aimed to assess the viability of a multivariate regression model utilizing ultrasound findings and serum markers for predicting thyroid cancer metastasis.

**Methods:**

A retrospective analysis of 98 thyroid patients admitted from January 2022 to October 2022 was conducted to categorize them into a metastasis group (n=20) and a non-metastasis group (n=78) based on postoperative pathological results. Both groups underwent ultrasound examination and serum marker testing. Correlative analysis was performed to explore the association between various indicators and thyroid cancer metastasis. A multivariate regression model was developed, and receiver operating characteristic (ROC) curves were used to assess the predictive value of ultrasound findings, serum markers, and their combination for thyroid cancer metastasis.

**Results:**

Statistically significant differences were found in the levels of ultrasound findings and serum markers between the two groups. Nodule boundaries, presence or absence of halos, margins, lobulation, capsular invasion, surface smoothness, nodule aspect ratio, uric acid, total cholesterol, triglyceride, and LDL cholesterol levels were predictors of metastasis in thyroid cancer. The AUC value of 0.950 for the prediction of thyroid cancer metastasis by ultrasound signs combined with serologic indicators was significantly higher than 0.728 and 0.711 predicted by ultrasound signs or serologic indicators alone.

**Conclusion:**

The multivariate regression model incorporating ultrasound findings and serum markers enhances the predictive accuracy for thyroid cancer metastasis, offering essential guidance for early prediction and intervention in a clinical setting.

## 1 Introduction

Thyroid cancer is a common malignant tumor in the endocrine system, ranking among the top in the incidence of malignant tumors in women in various countries (1). With the widespread use of techniques such as ultrasound examination and ultrasound-guided puncture, the incidence of thyroid cancer has significantly increased in the past few decades (2). Despite its slow clinical course and an overall 20-year survival rate of 90%, the disease-specific mortality is very low. However, local recurrence and distant metastasis are relatively common (3).

When fine-needle aspiration biopsy confirms malignant nodules in both lobes preoperatively, total thyroidectomy is usually performed, while thyroid lobectomy is typically conducted for cancer limited to one lobe (4). However, there is ongoing controversy regarding the necessity of performing thyroid lobectomy in patients with unilateral thyroid cancer accompanied by benign nodules on the contralateral side (5, 6). In summary, accurate identification of the pathological characteristics and lymph node metastasis of thyroid cancer preoperatively would contribute to precise treatment of thyroid cancer.

Research by Liu Y et al. (7) found that serum markers might serve as reliable predictive indicators of benign and malignant thyroid nodules. Furthermore, Zhao SS et al. (8) discovered that the aberrant expression of lipids and elevated levels of uric acid may potentially correlate with postoperative cervical lymph node metastasis in thyroid cancer patients. However, this conclusion has not been sufficiently validated in clinical practice, and relevant research is limited. Therefore, this study retrospectively analyzes the clinical data of a total of 98 thyroid patients admitted to the Third Affiliated Hospital of Qiqihar Medical University from January 2022 to October 2022, to investigate the feasibility of using a multivariate regression model incorporating ultrasound findings and serum markers to predict thyroid cancer metastasis.

## 2 Objectives and methods

### 2.1 Patient selection

A retrospective analysis was conducted on the clinical data of 98 thyroid cancer patients admitted to the third the Third Affiliated Hospital of Qiqihar Medical University from January 2022 to October 2022. Inclusion criteria were as follows: (1) patients with thyroid nodules that were firm, fixed, associated with cervical lymphadenopathy, or presenting compressive symptoms; (2) patients pathologically diagnosed with thyroid cancer; (3) patients undergoing initial surgical treatment for thyroid cancer; (4) patients with clear ultrasound images obtained preoperatively; (5) availability of complete clinical data.

Exclusion criteria were: (1) presence of concomitant malignant tumors; (2) patients with psychiatric disorders or cognitive impairments; (3) patients who received any radiotherapy before surgery; (4) thyroid cancer recurrence; (5) presence of non-solitary nodules; (6) patients with severe organ dysfunctions (heart, lung, liver, kidney, etc.); (7) presence of severe infectious or communicable diseases.

### 2.2 Ultrasonography examination

All patients underwent preoperative ultrasonography using the E9 ultrasound machine provided by the GE company, equipped with a linear array probe with probe frequencies ranging from 6 to 15 MHz. Patients were positioned supine on the examination table with the neck exposed. A pillow was placed under the patient’s neck to slightly elevate the chin and ensure relaxation of the neck muscles. Experienced ultrasound specialists performed two-dimensional ultrasound scans of the thyroid and cervical lymph nodes, observing and meticulously documenting specific sonographic features of the lesions and abnormal cervical lymph nodes from transverse to longitudinal views and storing the obtained images within the machine. Focus was placed on observing and recording features such as the position, maximum diameter, number, boundary, echogenicity, presence of peripheral halo, lobulation, capsular invasion, surface smoothness, echo texture, as well as the longitudinal-to-transverse ratio of thyroid cancer nodules. The aspect ratio of a nodule is calculated by dividing its longitudinal diameter by its transverse diameter. An aspect ratio > 1 typically indicates a malignant nodule, while an aspect ratio < 1 is more commonly associated with benign nodules.

### 2.3 Serum marker assessment

Preoperatively, all patients provided a fasting 5 mL serum sample, which, after centrifugation, was stored at -20°C until analysis. The serum samples were assayed using the ADVIA Centaur chemiluminescent system (Siemens/Bayer) to measure levels of thyroid-stimulating hormone, anti-thyroglobulin antibodies, and anti-thyroid peroxidase antibodies. Additionally, the BC-6800 automated hematology analyzer (Mindray Medical, Shenzhen) was used to evaluate platelet, leukocyte, neutrophil, lymphocyte, eosinophil, and monocyte levels. The BS-200 automated biochemical analyzer (Mindray Medical, Shenzhen) was employed to measure total bilirubin, direct bilirubin, indirect bilirubin, total protein, albumin, aspartate aminotransferase, glucose, blood urea nitrogen, creatinine, uric acid, triglycerides, total cholesterol, high-density lipoprotein cholesterol, and low-density lipoprotein cholesterol levels.

### 2.4 Postoperative occurrence of metastasis

The occurrence of cervical lymph node metastasis postoperatively was documented for all patients, with inclusion of 20 patients in the metastasis group (those presenting suspected metastatic lesions identified through CT and subsequently confirmed via pathological biopsy), and the remaining 78 patients in the non-metastasis group. Within the lymph node metastasis group, 8 patients had central lymph node metastasis, accounting for 40.00%, while 12 patients had lateral neck lymph node metastasis, accounting for 60.00%.

### 2.5 Statistical methods

Data were analyzed using SPSS version 25.0. Descriptive data were presented as counts and percentages [n(%)], and the χ2 test was employed. Normally distributed quantitative data were expressed as mean (± standard deviation) and analyzed using the *t*-test. Spearman’s correlation analysis was used for correlation assessments. Significant indicators between the two groups were determined, with a significance level set at α=0.05. Binary logistic regression analysis was performed, and receiver operating characteristic (ROC) curves were plotted to evaluate the value of ultrasound findings and serum markers, either separately or in combination, in predicting the risk of thyroid cancer metastasis. The predictive value was assessed using the area under the curve (AUC). A significance level of P < 0.05 was considered statistically significant.

## 3 Results

### 3.1 Comparison of demographic characteristics between the two groups

As depicted in **Table 1**, no statistically significant differences were observed in terms of gender, age, tumor location, and maximum diameter between the two groups (P > 0.05). However, there was a statistically significant difference in disease staging (P < 0.05), suggesting that gender, age, tumor location, and maximum diameter were unrelated to thyroid cancer metastasis. Conversely, the higher the disease stage of thyroid cancer patients, the more prone they were to develop metastasis.

**Table 1:** Comparison of demographic characteristics between the two groups.

Characteristic	Metastasis Group (n=20)	Non-Metastasis Group (n=78)	*χ^2^*	*P*
Gender				
Male	11 (55.00)	46 (58.97)	0.005	0.946
Female	9 (45.00)	32 (41.03)		
Age				
≥60 years	12 (60.00)	36 (46.15)	0.730	0.393
<60 years	8 (40.00)	42 (53.85)		
Tumor Location				
Upper lobe	4 (20.00)	12 (15.38)	1.012	0.798
Middle lobe	8 (40.00)	35 (44.87)		
Lower lobe	6 (30.00)	27 (34.62)		
Isthmus	2 (10.00)	4 (5.13)		
Maximum Diameter				
≥1	14 (70.00)	36 (46.15)	2.731	0.098
<1	6 (30.00)	42 (53.85)		
Disease Stage				
I	0 (0.00)	26 (33.33)	98.000	<0.001
II	0 (0.00)	52 (66.67)		
III	13 (65.00)	0 (0.00)		
IV	7 (35.00)	0 (0.00)		

### 3.2 Comparison of ultrasound findings between the two groups

As shown in **Table 2**, statistically significant differences were observed in various ultrasound indicators including nodule boundary, presence of halo, margins, lobulation, capsular invasion, and nodule aspect ratio (P < 0.05). The difference between the remaining indicators was not statistically significant (p > 0.05), suggesting a potential association between these ultrasound findings and thyroid cancer metastasis.

**Table 2:** Comparison of ultrasound findings between the two groups [n (%)].

Ultrasound Feature	Metastasis Group (n=20)	Non-Metastasis Group (n=78)	χ2	P
Nodule Boundary				
Clear	6 (30.00)	49 (62.82)	5.694	0.017
Indistinct	14 (70.00)	29 (37.18)		
Halo Presence				
Present	4 (20.00)	56 (71.79)	15.873	<0.001
Absent	16 (80.00)	22 (28.21)		
Margins				
Smooth	16 (80.00)	16 (20.51)	22.982	<0.001
Irregular	4 (20.00)	62 (79.49)		
Lobulation				
Present	11 (55.00)	5 (6.41)	24.069	<0.001
Absent	9 (45.00)	73 (93.59)		
Capsular Invasion				
Present	15 (75.00)	10 (12.82)	29.198	<0.001
Absent	5 (25.00)	68 (87.18)		
Echo Texture				
Homogeneous	6 (30.00)	21 (26.92)	0.000	1.000
Heterogeneous	14 (70.00)	57 (73.08)		
Nodule Aspect Ratio				
<1	3 (15.00)	40 (51.28)	7.100	0.008
≥1	17 (85.00)	38 (48.72)		

### 3.3 Comparison of serum markers between the two groups

As shown in **Table 3**, The differences in anti-thyroglobulin antibodies, neutrophils, lymphocytes, eosinophils, monocytes, total protein, aspartate aminotransferase, uric acid, total cholesterol, triglycerides, and LDL cholesterol were statistically significant (P < 0.05), and the differences in the remaining indices were not statistically significant (P > 0.05), which suggests that the above mentioned indices which are different may be associated with metastasis of thyroid cancer. may be associated with metastasis of thyroid cancer.

**Table 3:** Comparison of serum markers between the two groups.

Serum Marker	Metastasis Group (n=20)	Non-Metastasis Group (n=78)	t	P
Thyroid Stimulating Hormone (μU/mL)	3.59 ± 0.57	3.53 ± 0.61	0.414	0.682
Anti-Thyroglobulin Antibody (μU/mL)	173.28 ± 30.90	145.78 ± 34.87	3.457	0.002
Anti-Thyroid Peroxidase Antibody (μU/mL)	53.28 ± 5.61	53.99 ± 4.92	0.513	0.612
Platelets (×10^9/L)	284.82 ± 50.80	292.47 ± 57.56	0.585	0.563
Leukocytes (×10^9/L)	6.73 ± 0.94	6.50 ± 0.90	1.004	0.324
Neutrophils (×10^9/L)	4.05 ± 0.49	4.93 ± 0.85	6.027	<0.001
Lymphocytes (×10^9/L)	4.06 ± 0.67	1.58 ± 0.42	15.877	<0.001
Eosinophils (%)	47.10 ± 6.19	7.26 ± 1.28	28.617	<0.001
Monocytes (×10^9/L)	42.09 ± 4.03	0.85 ± 0.09	45.706	<0.001
Total Bilirubin (μmol/L)	24.70 ± 4.90	9.38 ± 1.05	16.610	<0.001
Direct Bilirubin (μmol/L)	4.04 ± 0.47	3.98 ± 0.43	0.458	0.650
Indirect Bilirubin (μmol/L)	3.75 ± 0.48	3.81 ± 0.48	0.488	0.629
Total Protein (g/L)	44.76 ± 5.18	47.89 ± 5.77	2.354	0.025
Albumin (g/L)	40.74 ± 6.51	41.49 ± 6.13	0.634	0.531
Aspartate Aminotransferase (u/L)	25.25 ± 5.10	22.58 ± 4.47	2.140	0.042
Blood Urea Nitrogen (μmol/L)	4.42 ± 0.79	4.67 ± 0.79	1.280	0.210
Creatinine (μmol/L)	45.66 ± 4.76	47.41 ± 4.31	1.490	0.148
Uric Acid (mmol/L)	286.38 ± 53.40	248.13 ± 47.90	3.112	0.002
Total Cholesterol (mmol/L)	7.17 ± 0.84	6.40 ± 0.98	3.533	0.001
Triglycerides (mmol/L)	2.84 ± 0.55	1.78 ± 0.45	7.869	<0.001
High-Density Lipoprotein Cholesterol (mmol/L)	1.65 ± 0.25	1.63 ± 0.41	0.254	0.801
Low-Density Lipoprotein Cholesterol (mmol/L)	4.50 ± 0.71	3.95 ± 0.59	3.166	0.004

### 3.4 Correlation of serum markers and thyroid cancer metastasis

As presented in **Table 4**, positive correlations were observed between nodule boundary, presence of halo, lobulation, capsular invasion, nodule aspect ratio, uric acid, total cholesterol, triglycerides, low-density lipoprotein cholesterol, and thyroid cancer metastasis, while negative correlations were evident for margins and thyroid cancer metastasis (P < 0.05). This suggests a significant association between the aforementioned ultrasound findings, serum markers, and thyroid cancer metastasis.

**Table 4:** Correlation between various indicators and thyroid cancer metastasis.

Indicator	Thyroid Cancer Metastasis	
	*r*	*P*
Nodule Boundary	0.267	0.008
Presence of Halo	0.428	<0.001
Margins	-0.511	<0.001
Lobulation	0.530	<0.001
Capsular Invasion	-0.575	<0.001
Nodule Aspect Ratio	0.295	0.003
Uric Acid	0.281	0.005
Total Cholesterol	0.313	0.002
Triglycerides	0.670	<0.001
Low-Density Lipoprotein Cholesterol	0.339	<0.001

### 3.5 Multivariate regression analysis of thyroid cancer metastasis

As outlined in **Table 5**, logistic regression analysis of the differing indicators revealed OR values >1 for nodule boundary, presence of halo, margins, lobulation, capsular invasion, surface smoothness, nodule aspect ratio, uric acid, total cholesterol, triglycerides, and low-density lipoprotein cholesterol levels, indicating that these ultrasound findings and serum markers are predictive factors for thyroid cancer metastasis.

**Table 5:** Multivariate regression analysis of thyroid cancer metastasis.

Indicator	Coef	Odds ratio	Lower ci	Upper ci	beta	P Value
Nodule Boundary	1.372	0.254	0.082	0.706	1.372	0.011
Presence of Halo	2.321	0.098	0.026	0.301	2.321	<0.001
Margins	2.741	15.500	4.933	60.189	-2.741	<0.001
Lobulation	2.882	17.844	5.312	68.790	-2.882	<0.001
Capsular Invasion	3.016	20.400	6.471	75.136	-3.016	<0.001
Nodule Aspect Ratio	1.786	0.168	0.037	0.548	1.786	0.007
Uric Acid	0.016	1.017	1.006	1.029	0.016	0.004
Total Cholesterol	0.906	0.404	0.209	0.714	0.906	0.003
Triglycerides	4.073	0.017	0.002	0.076	4.073	<0.001
Low-Density Lipoprotein Cholesterol	1.399	0.247	0.098	0.563	1.399	0.002

### 3.6 Predictive value of ultrasound findings, serum markers, or their combination for thyroid cancer metastasis

**Figure 1:**
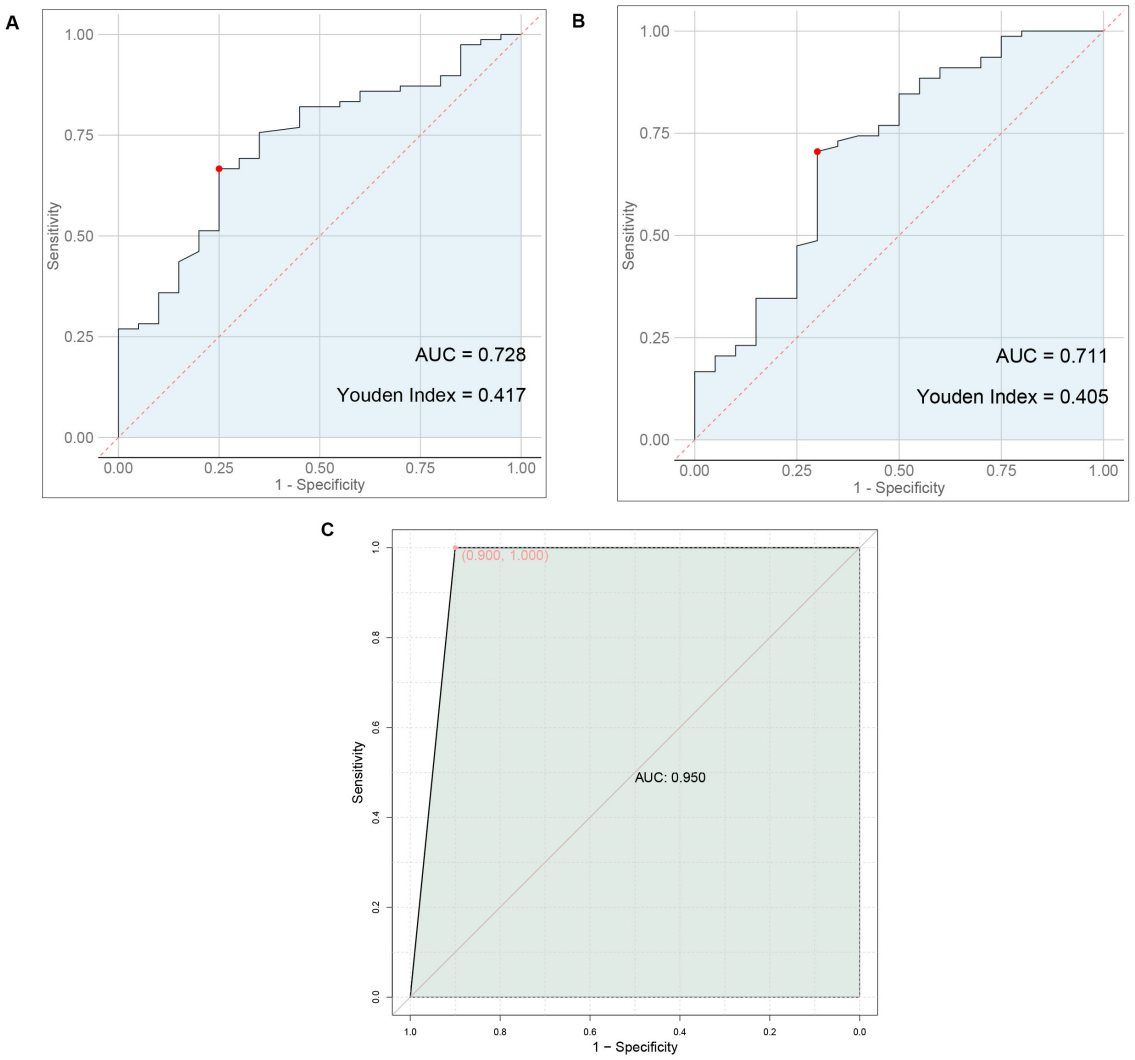
Predictive value of ultrasound findings, serum markers, or their combination for thyroid cancer metastasis. **(A)** ROC plot of ultrasound signs alone predicting thyroid cancer metastasis. **(B)** ROC plot of serum markers alone to predict metastasis in thyroid cancer **(C)** ROC plot of ultrasound signs combined with serologic markers to predict thyroid cancer metastasis.

As shown in **Figure 1**, ultrasound signs alone predicted thyroid cancer metastasis with an AUC value of 0.728, serologic markers alone predicted thyroid cancer metastasis with an AUC value of 0.711, and ultrasound signs combined with serologic markers predicted thyroid cancer metastasis with an AUC value of 0.950 that was significantly higher than that predicted alone.

## 4 Discussion

According to epidemiological surveys (9), the incidence of thyroid diseases in China has been increasing year by year. Related studies have pointed out (10, 11) that the high mortality rate of thyroid cancer may be related to disease metastasis. Metastasis not only affects the prognosis of patients, but also affects the selection of surgical methods. Therefore, the present study aimed to assess the ultrasound features of patients with thyroid cancer and perform serum marker detection in order to evaluate the risk of metastasis, propose appropriate and safe surgical strategies, and enhance patient prognosis. This holds significant clinical implications.

Clinically, preoperative ultrasound examination is the preferred method for determining whether thyroid cancer patients have metastasis. ZHAO SS et al.’s study (12) indicated that preoperative ultrasound examination is helpful to observe the metastatic status of patients. By in-depth exploration of the relationship between ultrasound features and clinical characteristics of thyroid cancer and cervical lymph node metastasis, it may have some value in evaluating whether thyroid cancer patients have metastasis. DAI Q et al. (13) found that the cervical lymph node metastasis rate of thyroid patients was 34.44%. In this study, the metastasis rate of thyroid patients was 20.41% (20/98), which was lower than the results of the above studies, possibly related to differences in age and other factors among the patients studied. Nodule boundary, presence of halo, margins, lobulation, capsular invasion, surface smoothness, and nodule aspect ratio are the main manifestations of ultrasound observation of thyroid cancer metastasis. The metastasis group showed a higher proportion of indistinct nodule boundaries, absence of halos around nodules, smooth edges, presence of lobulation, capsular invasion, and nodule aspect ratio ≥1 compared with the non-metastasis group (P<0.05), and this result was also confirmed in the subsequent correlation and binary logistic regression analyses, suggesting that combining the above ultrasound features can help determine whether thyroid cancer patients have metastasis. SU GY et al. (14) found that nodule diameter was also a risk factor for thyroid cancer metastasis, but this study did not find a relationship between maximum nodule diameter and thyroid cancer metastasis, possibly related to differences in sample size in different studies.

Previous study indicates (15, 16) that lipid abnormalities can lead to the signal transduction of tumor cells, promote the expression of adhesion molecules in vascular endothelial cells, and affect patients’ immune function, playing a direct or indirect role in the occurrence and progression of tumors. The formation of lipid rafts is closely associated with cholesterol, which can carry some cell membrane-related proteins and participate in the signal regulation of tumor cells (17, 18). Besides, previous studies have also shown (19) that overexpression of cholesterol in the body can activate the protein kinase B signaling pathway, thereby accelerating the growth of cancerous tissues and cells. Furthermore, there are reports indicating (20) that the growth of cancerous tissues is closely related to LDL-C, and about 75% of cholesterol in the body exists in the form of LDL-C. In addition, a study suggests (21) that stimulating vascular endothelial cells with LDL-C can increase the level of cell surface adhesion molecules, prolong the contact time of cancer cells in microvessels, and consequently increase the risk of metastasis. Similarly, literature points out (22, 23) that in cancer patients, intracellular TG is overexpressed during metastasis. This may be related to the following factors: 1) Elevated TG levels in the body can lead to insulin resistance in patients, increasing the risk of cell damage and thereby providing a pathway for tumor cell metastasis. 2) When tumor cells in the body metastasize, they require a large amount of lipids, which leads to lipid metabolism imbalance and the production of large amounts of cholesterol. 3) Elevated blood lipid levels meet the requirements of tumor cell metastasis, further accelerating tumor cell metastasis. Therefore, we speculate that TG, TC, and LDL-C may be involved in the process of metastasis in thyroid cancer patients. Furthermore, a study suggests (24) that serum uric acid has always been one of the prognostic factors for various cancers, such as nasopharyngeal and gastric cancers, and its expression in the body is closely related to inflammatory and oxidative stress reactions. Inflammatory and oxidative stress reactions can promote the extensive proliferation of cancerous cells, facilitate neovascularization, and thus provide opportunities for invasion and metastasis (25). This indicates that uric acid levels can also be used to assess the risk of metastasis in thyroid cancer patients. The results of this study show that the levels of TG, TC, LDL-C and uric acid in the metastasis group were higher than those in the non-metastasis group, consistent with the conclusions of the above studies. This further demonstrates that abnormal preoperative expression of TG, TC, LDL-C, and uric acid is associated with postoperative metastasis in thyroid cancer patients, a result that was confirmed in subsequent correlation and binary logistic regression analyses. This study proposes a predictive model combining ultrasound features and serum markers to assess the risk of metastasis in thyroid cancer patients. Traditional diagnostic methods often rely on single data sources, such as imaging examinations or blood tests alone. However, this study achieves a more accurate assessment of the likelihood of thyroid cancer metastasis by integrating both types of data. The AUC value of 0.950 for the prediction of thyroid cancer metastasis by ultrasound signs combined with serologic indicators was significantly higher than 0.728 and 0.711 predicted by ultrasound signs or serologic indicators alone. This suggests that preoperative ultrasound findings and serum markers can serve as predictive indicators for the risk of postoperative metastasis in patients. The combination of ultrasound findings with serum markers can synergistically increase the predictive value for the risk of postoperative metastasis.

Nonetheless, this study still has limitations, such as the relatively small sample size and the fact that the samples are all from a single hospital. Furthermore, the specific mechanisms by which ultrasound findings and serum markers lead to thyroid cancer metastasis require further in-depth analysis in the future.

In summary, the multivariate regression model combining ultrasound findings with serum markers can enhance the predictive value for thyroid cancer metastasis, thus guiding early prediction and intervention for the risk of thyroid cancer metastasis in clinical practice.

## Data Availability

The original contributions presented in the study are included in the article/supplementary material. Further inquiries can be directed to the corresponding author.
